# Editorial: It Is a Matter of Matters: Deciphering Structural and Functional Brain Connectivity

**DOI:** 10.3389/fnins.2022.951001

**Published:** 2022-06-16

**Authors:** Gopikrishna Deshpande, Vinoo Alluri, Aaryana Sharma, Madhura Ingalhalikar

**Affiliations:** ^1^Department of Electrical and Computer Engineering, AU MRI Research Center, Auburn University, Auburn, AL, United States; ^2^Department of Psychological Sciences, Auburn University, Auburn, AL, United States; ^3^Center for Neuroscience, Auburn University, Auburn, AL, United States; ^4^Alabama Advanced Imaging Consortium, Birmingham, AL, United States; ^5^Key Laboratory for Learning and Cognition, School of Psychology, Capital Normal University, Beijing, China; ^6^Department of Psychiatry, National Institute of Mental Health and Neurosciences, Bangalore, India; ^7^Centre for Brain Research, Indian Institute of Science, Bangalore, India; ^8^Cognitive Science Laboratory, Indian Institute of Information Technology, Hyderabad, India; ^9^Symbiosis Center for Medical Image Analysis, Symbiosis International University, Pune, India

**Keywords:** functional MRI (fMRI), functional connectivity, structural connectivity, brain connectivity, mental disorders

This special issue highlights state-of-the-art quantitative techniques to translate the relation between structural and functional brain connectivity, or jointly analyze brain structure and function to understand the aberrations in neurological diseases and neuropsychiatric disorders. The technical advancements in multimodal imaging technology for investigating brain structure and function are succinctly summarized by Venkadesh and Van Horn in “Integrative Models of Brain Structure and Dynamics: Concepts, Challenges and Methods”. This paper introduces theoretical frameworks to interpret empirically measured brain connectivity including the graph data structure that can be used to indicate connections between regions as well as analyze local and global properties, as well as modularity to characterize independent and dependent communities of neural networks. Next, they dwell on mechanisms acting on multiple timescales that govern the reciprocal relationship between neural network structure and its intrinsic dynamics with special focus on the macroscopic scale. It was concluded that multimodal imaging provided a more accurate depiction of structure-function relationships in the brain.

Structure-function relationships were further analyzed by Safai et al. in “Multimodal Brain Connectomics-Based Prediction of Parkinson's Disease Using Graph Attention Networks” with translation to Parkinson's disease (PD). The paper illustrates why multimodal features are beneficial in analyzing structure-function connections and predicting PD. The authors use a graph attention network based deep learning classification framework with feature sets derived from fMRI-based functional connectivity, diffusion MRI based structural connectivity as well as a combination of both. They show that the multimodal feature set outperforms models based on unimodal features for predicting PD. The GAT architecture also allowed them to outperform previous classification models of PD based on brain imaging data, highlighting the utility of deep learning.

Wang et al. bring in an interesting perspective about the utility of functional connectivity derived from white matter, which is traditionally used to investigate structural connectivity, in their paper titled “Altered Homotopic Functional Connectivity Within White Matter in the Early Stages of Alzheimer's Disease”. The authors combined resting state fMRI with diffusion tensor imaging (DTI) so that they could point to an anatomic basis for altered white matter homotopic functional connectivity that they observed in early stages of Alzheimer's disease. The study demonstrates that it is possible to imagine other uses for a given modality than is traditionally used for. Further, investigation of structure-function relationships in the brain may not be boxed into data fusion of fMRI and DTI modalities, but rather also be investigated by using the functional signal available in diffusion MRI. This provides a new perspective for multimodal data fusion.

In their paper titled “Assessment of Disrupted Brain Structural Connectome in Depressive Patients with Suicidal Ideation Using Generalized Q-Sampling MRI,” Chen et al. provide an illustration of how structural connectivity investigations have translational potential in the clinical. They investigated structural alterations in depressed individuals with and without suicidal ideation as well as healthy controls. It was observed that the generalized fractional anisotropy value showed differences in particular regions of the brain such as the corpus callosum and anterior cingulate. Changes in the corpus callosum is known to affect emotional processing, working memory and cognitive control. Similarly, the cingulate gyrus regulates emotions. Network-based statistics analysis indicated that interconnections between these subnetworks were significantly weaker in depressed individuals with suicidal ideation as opposed to both the control groups. This enriches our understanding of suicidal ideation in terms of concomitant structural connectivity alterations in the brain and provides therapeutic targets that can be pursued in future studies.

Likewise, the translational potential of functional connectivity is demonstrated by Fu et al. in their paper titled “Whole-Brain Functional Network Connectivity Abnormalities in Affective and Non-Affective Early Phase Psychosis.” They investigate both static and dynamic functional connectivity abnormalities in early phase of psychosis, a time period when intervention may be most effective. They also use these tools to identify commonalities and differences between affective and non-affective psychosis. These findings have a significant bearing on future studies which may devise therapies targeting early psychosis.

In their review titled “Altered Structural and Functional MRI Connectivity in Type 2 Diabetes Mellitus Related Cognitive Impairment: A Review,” Lei et al. integrate known findings of alterations in neuroplasticity and energy metabolism in Type-2 Diabetes with those reflected in structural and functional brain connectivity derived from MRI. The paper proposes brain connectivity to be the link between neural plasticity and energy metabolism. Specifically, they hypothesize that diabetes may lead to abnormalities in neuroplasticity and energy metabolism in the brain through multiple pathological pathways, ultimately leading to cognitive impairment that may reflect to MRI structural connectivity and functional connectivity, respectively. They also integrate our knowledge of pathological processes underlying Alzheimer's disease (such as Aβ) with those underlying diabetes. Their review illustrates how functional and structural brain connectivity may provide pathways to understand existing mechanistic models of altered brain function in disorders.

In a basic science application with potential translation to the clinic, Robinson et al. investigate structure-function correspondence in the hippocampus (a structure critical in many brain disorders) in their paper titled “Neurofunctional Segmentation Shifts in the Hippocampus”. The study addresses how to reconcile disparate findings of structural and functional organization within the hippocampus, with cytoarchitecture favoring dorsal-ventral layering while neurofunctional topography supporting anterior-posterior and medial-lateral functional differentiation. The uniqueness of the study lies in the fact that it uses high resolution MRI data acquired at 7T and uses metanalytic connectivity modeling in addition to traditional measures of connectivity. The study suggests that cognitive processing and stimulus features may influence how the hippocampus activates, and reflects a further need to study the functional properties of the hippocampus using a wide variety of stimuli in order to infer structure-function correspondence in the hippocampus. This may be critical for understanding the basis of hippocampal involvement in disorders such as post-traumatic stress disorder and schizophrenia.

Finally, Shi et al. provide an illustration of the use of a non-MRI modality such as functional near infrared spectroscopy (fNIRS) to understand bran function. The authors evaluate the response of the cerebral cortex to different upper limb movement patterns. Such processes are difficult to study using MRI given its restrictive environment. The study highlights the role of non-MRI modalities in investigating brain structure and function.

In conclusion, the special issue highlights many methodological advances in investigating brain structure, function and their inter-relationships using brain connectivity, reviews prior literature on these topics and illustrates the utility of such methods in understanding brain function in both health and disease.

## Author Contributions

GD, VA, and MI edited this special issue. AS and GD contributed to writing the first draft of the editorial. VA and MI made edits to the manuscript. All authors contributed to the article and approved the submitted version.

## Conflict of Interest

The authors declare that the research was conducted in the absence of any commercial or financial relationships that could be construed as a potential conflict of interest.

## Publisher's Note

All claims expressed in this article are solely those of the authors and do not necessarily represent those of their affiliated organizations, or those of the publisher, the editors and the reviewers. Any product that may be evaluated in this article, or claim that may be made by its manufacturer, is not guaranteed or endorsed by the publisher.

